# Streptozotocin-Induced Diabetes Models: Pathophysiological Mechanisms and Fetal Outcomes

**DOI:** 10.1155/2014/819065

**Published:** 2014-05-27

**Authors:** D. C. Damasceno, A. O. Netto, I. L. Iessi, F. Q. Gallego, S. B. Corvino, B. Dallaqua, Y. K. Sinzato, A. Bueno, I. M. P. Calderon, M. V. C. Rudge

**Affiliations:** ^1^Laboratory of Experimental Research on Gynecology and Obstetrics, Graduate Program in Gynecology, Obstetrics and Mastology, Botucatu Medical School, UNESP-Universidade Estadual Paulista, Distrito de Rubião Júnior S/N, 18618-970 Botucatu, SP, Brazil; ^2^Department of Gynecology and Obstetrics, Botucatu Medical School, UNESP-Univsidade Estadual Paulista, Distrito de Rubião Júnior S/N, 18618-970 Botucatu, SP, Brazil

## Abstract

Glucose homeostasis is controlled by endocrine pancreatic cells, and any pancreatic disturbance can result in diabetes. Because 8% to 12% of diabetic pregnant women present with malformed fetuses, there is great interest in understanding the etiology, pathophysiological mechanisms, and treatment of gestational diabetes. Hyperglycemia enhances the production of reactive oxygen species, leading to oxidative stress, which is involved in diabetic teratogenesis. It has also been suggested that maternal diabetes alters embryonic gene expression, which might cause malformations. Due to ethical issues involving human studies that sometimes have invasive aspects and the multiplicity of uncontrolled variables that can alter the uterine environment during clinical studies, it is necessary to use animal models to better understand diabetic pathophysiology. This review aimed to gather information about pathophysiological mechanisms and fetal outcomes in streptozotocin-induced diabetic rats. To understand the pathophysiological mechanisms and factors involved in diabetes, the use of pancreatic regeneration studies is increasing in an attempt to understand the behavior of pancreatic beta cells. In addition, these studies suggest a new preventive concept as a treatment basis for diabetes, introducing therapeutic efforts to minimize or prevent diabetes-induced oxidative stress, DNA damage, and teratogenesis.

## 1. Introduction

*Diabetes mellitus* (DM) is a chronic disease characterized by hyperglycemia resulting in insulin resistance and/or insulin secondary deficiency caused by the failure of beta- (*β*-) pancreatic cells. Diabetes can be classified into four clinical categories, type 1 diabetes (due to autoimmune destruction of the *β* cells, usually leading to absolute insulin deficiency), type 2 diabetes (due to a progressive insulin secretory defect in the background of insulin resistance), gestational* Diabetes mellitus* (GDM) (diabetes diagnosed during pregnancy that is not clearly overt diabetes), and other specific types of diabetes due to other causes, for example, genetic defects in *β* cell function or insulin action, drug- or chemical-induced alterations (such as in the treatment of HIV/AIDS or after organ transplantation), and any diseases of the exocrine pancreas characterized by a process that diffusely injures the pancreas can cause diabetes. Diabetes is usually diagnosed based on plasma glucose criteria, either the fasting plasma glucose (FPG) or the 2 h plasma glucose (2 h PG) value after a 75 g oral glucose tolerance test (OGTT). Besides, recently, an International Expert Committee added the A1C (threshold ≥ 6.5%) as a third option to diagnose diabetes. In type 1 diabetes, patients often present acute symptoms of diabetes and markedly increased glucose levels and in some cases ketoacidosis. Type 2 diabetes is frequently not diagnosed until complications appear. ADA for the first time recommended that all pregnant women not known to have prior diabetes undergo a 75 g OGTT at 24–28 weeks of gestation based on an International Association of Diabetes and Pregnancy Study Groups (IADPSG) consensus meeting. In U.S. approximately one-fourth of the population may have undiagnosed diabetes. Acquired processes include pancreatitis, trauma, infection, pancreatectomy, and pancreatic carcinoma (such as cystic fibrosis). However, for the clinician and patient, it is less important to label the particular type of diabetes than it is to understand the pathogenesis of hyperglycemia and to treat it effectively [1].

Research in health has been improving the quality of medical care, influencing health policies and ensuring patient safety. Translational research is an important tool that allows researchers in clinical practices to establish knowledge and implement the results [2]. Translational research covers two areas. One is the process of applying discoveries generated during research in the laboratory and in preclinical studies to the development of trials and studies in humans. The second area of translation concerns research aimed at enhancing the adoption of best practices in the community. The cost-effectiveness of prevention and treatment strategies is also an important part of translational science [3]. According to this definition, translational research is part of a unidirectional continuum in which research findings move from the researcher's bench to the patient's bedside and the community. In the continuum, the first stage of translational research (T1) transfers knowledge from basic research to clinical research, while the second stage (T2) transfers findings from clinical studies or clinical trials to practice settings and communities where the findings improve health [4]. Due to ethical issues involving human studies that can require invasive aspects and the multiplicity of uncontrolled variables that can alter the uterine environment during clinical studies [5], it is necessary to use animal models to better understand diabetic pathophysiology [6]. Thus, this review aimed to gather information about pathophysiological mechanisms and fetal outcomes in streptozotocin-induced diabetic rats.

## 2. Pancreatic Islets: Structure and Function

The pancreas is a complex organ that consists of two functionally and morphologically distinct cell populations derived from the endoderm. The exocrine pancreas consists of acinar cells that secret digestive enzymes, such as amylases, lipases, proteases, and nucleases, which are emptied into the pancreatic duct through an elaborately branched network of tubules composed of epithelial cells. Acinar cells also produce bicarbonate ions and electrolytes, which, together with exocrine enzymes, are transported through the main duct into the duodenum, where they contribute to food processing [7, 8].

Groups of endocrine cells called pancreatic islets represent the endocrine portion, which composes only approximately 2% of the pancreas. Each islet is composed of at least five types of cells, including insulin-producing *β* cells (65–80%) [9], glucagon-releasing *α* cells (15–20%) [10], somatostatin-producing *δ* cells (3–10%) [11], pancreatic polypeptide-containing PP cells (1%) [12], and ghrelin-containing *ε* cells [13]. All of these hormones are involved in the regulation of nutrient metabolism and glucose homeostasis [14]. The cytoarchitecture of rodent and human islets presents notable differences [15, 16]. In islets from mice and other rodents, the *β* cells are predominately located in the central core with *α* and *δ* cells localized in the periphery forming a mantle [17–21]. In human and monkey islets, *α* cells are not localized in the periphery but rather are dispersed throughout the islet [15, 16, 19, 21, 22].

There are several lines of evidence that pancreatic islets cannot be considered aggregates of cells. It was well established more than 20 years ago that the integrated secretory responses of isolated islets are greater than those of dispersed islet cells, suggesting that cell-to-cell interactions are necessary for the normal secretory function of the endocrine pancreas [23–27].

However, there are no reports regarding the effect endocrine hormones in the diabetic environment. Several studies have emphasized the importance of interactions among the different cells [28, 29] and between cells of the same type [29, 30] in metabolic control. In normal islets, the insulin-producing cells are under the influence of other hormone-secreting islet cells. Glucagon is known to enhance both insulin and somatostatin secretion, while somatostatin exerts an inhibitory effect on insulin and glucagon secretion [31, 32]. Communication among these hormones is important to maintain physiological balance, and any disorder in this system would result in excessive blood glucose levels, for example, damaging the organism.

Hyperglycemia during pregnancy impairs the intrauterine environment, affecting normal fetal development and resulting in long-term effects on the function and structure of fetal pancreatic islets [33, 34]. This status increases the offspring's risk of obesity/adiposity, glucose intolerance, and type 2 diabetes later in life [1, 35–37]. Animal studies have shown that the offspring of diabetic rats can be insulin resistant [38, 39] and diabetic [39, 40]. Studies support the concept that developing organs have critical periods of intense structural and functional reorganization. In the case of the pancreas, this circumstance may render it vulnerable to environmental stimuli [41, 42], which may lead to consequences for the next generation [43] and future studies should consider the hormone interactions involved for this glucose control.

The literature shows that the administration of pancreatic hormones analogs (insulin, glucagon, and somatostatin) in in vitro studies is important to investigate the mechanisms of hormonal synthesis and secretion in an isolated manner [44–46]. In addition, insulin is the most studied hormone in the maternal and fetal organism in an attempt to understand the repercussions of hyperglycemia [47–54]. In our laboratory, we hypothesized that glucoregulatory hormones such as glucagon and somatostatin, in addition to insulin, are relevant for embryo-fetal development and diabetes-derived alterations. We performed an experimental study in rats to evaluate the importance of the endocrine pancreatic hormonal triad in maternal, fetal, and neonatal organisms exposed to a hyperglycemic intrauterine environment. According to our results, somatostatin levels were altered in all developmental points studied, showing that pancreatic alteration in maternal and fetal organisms persisted in the neonatal period. These results suggest that somatostatin might be a predictor of adverse effects in adulthood. In fact, our data show the importance of studying hormonal interactions in the endocrine pancreas to understand the pathophysiological mechanisms related to glycemic control in maternal and fetal organisms [55].

## 3. Pathophysiological Mechanisms of Diabetic Disease

The chronic hyperglycemia of diabetes is associated with long-term damage, dysfunction, and failure of different organs, especially the eyes, kidneys, nerves, heart, and blood vessels [1]. Type 1* Diabetes mellitus* results from the cell-mediated autoimmune destruction of the *β*-cells of the pancreas. Markers of the immune destruction of the *β*-cell include islet cell autoantibodies, autoantibodies to insulin, autoantibodies to GAD (GAD65), and autoantibodies to the tyrosine phosphatases IA-2 and IA-2b [1]. Autoimmune destruction of *β*-cells has multiple genetic predispositions and is also related to environmental factors that are still poorly defined. Individuals who present Type 2* Diabetes mellitus* have insulin resistance and usually develop relative (rather than absolute) insulin deficiency. Although the specific etiologies are not known, autoimmune destruction of *β*-cells does not occur. Most patients with this form of diabetes are obese, and obesity itself causes some degree of insulin resistance [1]. Elevations in plasma glucose and free fatty acids are thought to increase reactive oxygen species (ROS) levels [56, 57], which in turn activate inflammation signaling pathways such as mitogen-activated protein kinases [58] and nuclear factor-kB [59]. The activation of these inflammation cascades is thought to cause insulin resistance [60].

Gestational* Diabetes mellitus* (GDM) has been defined as any degree of glucose intolerance with onset or first recognition during pregnancy (ADA). Glucose intolerance was first introduced in 1979 to replace “borderline” diabetes and other categories of hyperglycemia that did not appear to carry a risk of microvascular complications [61, 62]. It was only in the most recent reports that the category of nondiabetic fasting hyperglycemia was defined and given the name impaired fasting glycemia (IFG) [63, 64]. This indicates glucose concentrations that are clearly above normal but fall short of the diagnostic value for diabetes [65].

## 4. Diabetes and Pregnancy: Experimental Models

Experimentally induced diabetes through the administration of *β*-cytotoxic drugs such as streptozotocin (STZ) is well characterized [66]. Streptozotocin is an antimicrobial agent and has also been used as a chemotherapeutic alkylating agent [67]. “Streptozotocin diabetes” [68] is caused by the specific necrosis of the pancreatic *β*-cells, and this agent is the first choice for diabetes induction in animals [69, 70]. Depending on the animal strain, dose, route of drug administration, and the life period in which STZ is administered in rats, severe diabetes (blood glucose superior to 200/300 mg/dL) [71–77] or mild diabetes (glycemia between 120 and 200/300 mg/dL) are generated [68, 78–81]. For severe diabetes induction, STZ is administered at 40–50 mg/kg body weight intravenously or intraperitoneally during adulthood. After approximately three days, these animals present blood glucose levels greater than 300 mg/dL [79, 82–86]. In our laboratory, to induce mild diabetes, which is characterized by low glycemic intensity, the rats received a STZ injection (dose of 100 mg/kg body weight) subcutaneously at birth. Approximately three days after STZ administration, these animals developed hyperglycemia (>300 mg/dL) and presented low blood glucose levels (120–200 mg/dL) at adulthood [83, 85–93]. This fact might be explained by the high regenerative capacity of *β*-cell during the neonatal period [94, 95].

The literature has shown that several organs are able to undergo catch up growth when necessary [96]. In case of severe cell loss or physiological conditions, the pancreatic *β*-cells of rodents can regenerate in the early life period [97]. Cell regeneration can occur through different mechanisms such as neogenesis, proliferation [98, 99], and transdifferentiation [100]. Scaglia et al. [101] showed that during the neonatal period, the pancreas suffers physiological changes, events that can also be identified in other organs, for example, liver, kidneys, and central nervous system [102–105]. This pancreatic remodeling is due to increased replication and apoptosis rates of *β*-cells between days 13 and 17. These data show that in physiological conditions the organism has a dynamic *β*-cell mass, maintaining glucose homeostasis [95, 106].

Because *β*-cells are able to regenerate in physiological conditions, the next step was to develop an experimental model to induce islet injury to study the mechanisms involved in cell regeneration process. Bonner-Weir et al. [97] published some of the first data about pancreatic islet regeneration, administrating STZ on the second postnatal day. Two days after the induction of diabetes, the animals presented high blood glucose levels (>300 mg/dL) and reduced *β*-cell numbers compared to the control group. At postnatal day 10, the animals became euglycemic, and partial regeneration of pancreatic *β*-cells was evidenced. The authors suggested cellular proliferation as the mechanism of cell regeneration. After STZ administration, cells that were not affected by STZ-induced necrosis showed increased mitotic characteristics. Bonner-Weir et al. [107] showed increased mitosis, apoptosis, and hypertrophic cells and suggested that hypertrophy might be related to increased *β*-cell mass given that cell death is a mechanism of regulation related to the rate of mitosis, which could maintain an appropriate number of islet cells. Therefore, according to these authors, the increased *β*-cell mass could be due to replication, individual cell hypertrophy, or islet neogenesis by ductal cell differentiation [108]. Regarding the regeneration mechanisms of *β*-cells, some authors also suggest cell transdifferentiation from non-*β*-cells to insulin-producing cells. In contrast, Scaglia et al. [101] concluded that, once cells do not present hormonal co-expression, there is no transdifferentiation, suggesting that non-*β*-cells are not differentiating into *β*-cells.

In contrast, other authors defend the idea that *α*-cells are able to differentiate into *β*-cells by direct conversion of transcription factors [100, 109–112]. Some of the essential transcription factors involved in pancreatic regeneration have been investigated, such as neurogenin 3 (Ngn3), paired domain homeobox gene 4 (Pax4), and homeobox-containing gene (Arx). Ngn3 is expressed by precursors of the endocrine pancreas, Pax4 has selective expression throughout the pancreatic islet during its development and is then restricted to *β*-cells [113, 114], and Arx is expressed by pancreatic *α*-cells [100].

Liang et al. [95] found that *β*-cells were damaged four days after neonatal STZ induction. At day 8, these authors verified that there was *β*-cell recuperation, but 20 days after STZ injection the *β*-cell mass was still reduced, even though blood glucose levels reverted to normal. This study focused on transcription factors, and the authors used double immunofluorescence to stain Ngn3 and insulin or glucagon. By analyzing the coexpression of Ngn3 and glucagon, they observed abundant expression of Ngn3 in the *α*-cells of STZ-treated rats 8 and 12 days after STZ injection. However, in the control rats, few *α*-cells expressed Ngn3. The results of the diabetic group indicated that *α*-cells dedifferentiated into precursor cells and may be candidates for *β*-cell formation. The coexpression of Pax4 and insulin or glucagon was also studied and indicated a relationship between insulin and Pax4 in both the control and STZ groups. However, the coexpression of Pax4 and glucagon was verified 12 and 20 days after STZ administration. Ngn3 expression is necessary for transdifferentiation from *α*- to *β*-cells. In addition, Pax4 is an important transcription factor that specifies the *β*-cell lineage. The same authors observed Pax4 expression in *α*-cells of both control and STZ-treated rats, but this coexpression increased in the *α*-cells at day 20. These results demonstrate that *α*-cells are sources of *β*-cell regeneration and can undergo transdifferentiation. Another study using STZ showed that Arx inactivation in *α*-cells results in pancreatic islet hypertrophy and increased number of cells that are phenotypically *β*-cells. The authors suggest that when *α*-cells are subjected to Arx inactivation, they undergo transdifferentiation to *β*-cells. These results show that strategies aiming at inhibiting the expression of Arx may offer new avenues for diabetes treatment [115].

Therefore, the literature includes several mechanisms to explain *β*-cell regeneration, and most studies suggest that the proliferation of the remaining *β*-cells is the primary source of regeneration. Nevertheless, current studies have demonstrated the direct participation of *α*-cells in *β*-cell regeneration. Thus, we conclude that the regeneration of pancreatic *β*-cells may be due to different mechanisms, but further studies are needed to precisely elucidate each mechanism and its contribution to the regeneration as a whole.

## 5. Diabetes-Induced Teratogenesis

Diabetes has been recognized as a disease that increases the risk of birth defects in offspring by 3 to 5 times [116]. A significant improvement has been observed in the evolution of the diabetic pregnancy after the discovery of insulin, reducing the incidence of ketoacidosis, spontaneous abortions, stillbirths, and congenital malformations [117]. A total of 25% of offspring have been reported presenting these complications, and early detection and subsequent strict metabolic control of pregnant women with diabetes should decrease the frequency and severity of some of these complications in offspring [118]. Studies have shown that spontaneous abortions can result from the malformation of structures required for fetal viability, such as the cardiovascular system or the placenta, but could also be attributable to maternal effects, such as endocrinopathies or vascular complications affecting uterine perfusion [119].

The following are the two principal advances that have improved the offspring survival rate during diabetic pregnancy: (1) a good maternal glycemic control to reduce the morbidity and mortality of both the mother and fetus/neonate [120]; (2) availability of surfactant to reduce perinatal mortality from respiratory distress syndrome (RDS) [121]. Uncontrolled diabetic status throughout the pregnancy has been associated with a spectrum of disorders involving neural tube defects (NTDs), including spina bifida, anencephaly, encephalocele, holoprosencephaly, and cardiovascular [122–124] kidney, and skeletal system defects in addition to growth delay and miscarriage [116]. In fact, any organ can be affected, and 8% to 12% of diabetic pregnant women presented malformed fetuses [125].

Experimental studies have also been performed to understand diabetic teratogenesis. Damasceno et al. [126] and Volpato et al. [75] administered STZ (40 mg/kg) to adult virgin female Wistar rats before mating. During pregnancy, these rats presented hyperglycemic levels higher than 200 mg/dL. At term, the fetuses from diabetic dams presented skeletal (nonossified sternebrae and cleft palate) and visceral malformations (microphthalmia and hydronephrosis). Gäreskog et al. [127], using a similar diabetes model, recovered embryos from diabetic rats at day 10 or 11 of pregnancy. These embryos were cultured within their intact visceral yolk sac for 24 or 48 h and presented decreased Bcl-2 levels and increased Bax levels and increased activation of caspase 3. Thus, exposure to diabetes during organogenesis increased cellular apoptosis and embryonic dysmorphogenesis. However, some skeletal defects that can occur in human diabetic pregnancy, particularly caudal regression syndrome, are rarely observed in animal models, making it difficult to study their molecular etiology [119].

Several studies have tried to identify biochemical disturbances associated with malformations in animal models of diabetic pregnancy. Teratogenic processes in embryonic tissues include alterations of metabolic and signaling systems [118] such as metabolism of inositol [128], the polyol pathway [129], arachidonic acid/prostaglandins [130, 131], and reactive oxygen species (ROS) [132]. In the polyol pathway, the aldose reductase enzyme is responsible for catalyzing excess glucose into sorbitol. Sorbitol accumulation has been demonstrated to negatively affect cell function in glucose-permeable tissues. However, aldose reductase inhibitors (ARIs) can diminish some diabetes-related changes in affected tissues without modifying the hyperglycemia. It has been proposed that polyol pathway overactivity is responsible for diabetic nephropathy, neuropathy, and retinopathy due to the depletion of myoinositol, and this could be applied to diabetic congenital malformations [118].

Another theory centers on linoleic acid. It is the precursor of arachidonic acid, an essential fatty acid required throughout gestation [133]. The literature shows that arachidonic acid release from plasma membranes by phospholipase A2 is lower in diabetic rodents. The formation of the palate, the neural tube, the heart, and external genitalia involve the folding and fusion of opposing layers and require phosphatidylinositol turnover and arachidonic acid signaling [131]. Several studies have identified PGE2 as a prostaglandin (PG) derived from arachidonic acid involved in the prevention of malformations in experimental diabetic models [134, 135]. Supporting this idea, one study showed that the concentration of PGE2 decreased during neurulation in embryos from a diabetic mouse [136]. In vitro as well as in vivo results demonstrated that a high glucose concentration causes decreased cyclooxygenase (enzyme catalyzing the synthesis of PGE2 from arachidonic acid) gene expression [137], suggesting that diabetes causes decreased prostaglandin biosynthesis and that the inhibition of the arachidonic cascade may be a cause of diabetic embryopathy [138].

Another hypothesis is that increased glucose metabolism enhances the production of ROS, causing oxidative stress [139]. In the embryo, the energy metabolism is characterized by a high rate of glycolysis and lactic acid production (anaerobic glycolysis) with minimal activity of the Krebs cycle-electron transport system [138]. In accordance with the low activity of the mitochondrial oxidative pathway, scavenging enzymes such as superoxide dismutase (SOD), catalase, and glutathione peroxidase (GPx) seem to be immature during the period of early organogenesis [140]. A study performed on cultured rat embryos in high glucose (25 and 50 mM glucose) showed increased activity of the free radical scavenging enzyme superoxide dismutase (SOD) providing evidence of enhanced ROS production in a hyperglycemic environment [141]. Kinalski et al. [142] verified an increase in malondialdehyde (MDA) levels and reduced glutathione (GSH) and decreased activity of cytoplasmic Cu/Zn superoxide dismutase (Cu/Zn SOD) in the infants of mothers with pregestational and gestational diabetes. These data show increased oxidative stress and lipid peroxidation in these fetuses, which serve as indicators of fetal distress caused by maternal hyperglycemia [143].

Wentzel and Eriksson [144] evaluated embryoneural crest cells recovered from inbred Sprague-Dawley rat exposed to 5.5 or 30 mmol/L glucose for 48 hr on gestational day 10. Cells exposed to 30 mmol glucose/L presented decreased mRNA levels of catalase, Cu/Zn SOD, manganese superoxide dismutase, and extracellular superoxide dismutase. This altered gene expression induced by glucose may be the etiology of malformations in diabetic pregnancy.

## 6. Diabetes-Induced Oxidative Stress and DNA Damage

In addition to reactive oxygen and nitrogen species, the products of free radicals, which are dependent on fatty acid oxidation, can induce chromosome breaks [145, 146]. Therefore, these products can interact with the embryo chromatin, resulting in congenital malformations [147]. Free radicals can also react with DNA bases, impairing their structure [148, 149] and potentially leading to mutations [148–150]. DNA oxidation is the most common type of damage [151], although the methods used to assess this damage are still controversial. One marker used to study oxidative DNA damage [152, 153] is 8-OHdG or 8-oxo-7,8-dihydro-2-deoxyguanosine (8-oxodGuo or 8-oxoGua). It is a product of the deoxyguanosine nucleoside oxidation that is directly excreted in urine. Qiu et al. [154] demonstrated that the 8-OHdG urine concentration might be related to increased risk for gestational diabetes mellitus.

To evaluate DNA damage levels, the comet assay presents advantages compared to other methods to detect genotoxic substances. This test is not useful for detecting mutations but can detect genomic lesions, which can result in mutation. In contrast to mutations, the genomic lesions detected by the comet assay can be repaired. The comet assay is fast and sensitive; moreover, it can detect oxidized DNA bases using endonucleases such as endonuclease III (Endo III) and formamidopyrimidine DNA glycosidase (Fpg). Use of Fpg and Endo III allows the identification of both oxidized purine and pyrimidine bases, respectively [155, 156].

Although streptozotocin is an alkylating agent, it is also useful in genotoxicity studies. Some authors have suggested that STZ can irreversibly damage *β*-cell DNA. To investigate this hypothesis, Mossman et al. [157] performed an in vitro study showing that STZ induces single-strand DNA breaks in rodent cells (RINr 38), and these lesions are repaired 24 hours after STZ exposition. Studying the same cell lineage, Pettepher et al. [158] demonstrated that STZ also induces alkali-labile site breaks in mitochondrial DNA, and even though the formation of this lesion is dose-dependent, it can be repaired as well. After 8 hours of STZ exposition, 55% of the mitochondrial DNA lesions were repaired, rising to 70% in 24 hours. These data confirm that STZ by itself is not responsible for the high levels of DNA damage.

In regard to experimental studies with mild and severe diabetes, Lima et al. [86] evaluated oxidative damage in the lymphocytes of pregnant diabetic rats and whole blood samples of their offspring by the comet assay using repair enzymes (Endo III and Fpg). These authors found that mildly diabetic rats and their offspring presented more sensitive sites to Fpg, reflecting damage related to hyperglycemia. Tats with severe diabetes and their offspring showed oxidative DNA damage detected by Fpg as well as by Endo III, typical general diabetes outcomes. The enzymatic indication of DNA damage suggests that the repercussions of maternal diabetes are associated with oxidative lesions in maternal and fetal DNA. Damasceno et al. [159] demonstrated that severely diabetic rats presented higher DNA damage levels at term pregnancy compared to control rats. In addition, their offspring also showed higher DNA damage levels and increased rate of congenital malformations at term, confirming the interaction between hyperglycemia-induced genotoxicity and teratogenesis. These studies show the relationship among diabetes, oxidative stress, and oxidative DNA damage.

Although many studies have been performed to understand the congenital malformations induced by diabetes, additional research is necessary to identify new markers involved in the regulation of embryogenesis and occurrence of congenital malformations. It is necessary to comprehend DNA damage trigger factors in diabetes to reduce the impairment of gene expression, avoid fetal congenital malformation, and contribute to the normal development of organs during organogenesis.

## 7. Conclusion

Pancreatic islet loss and reduction of insulin-producing beta cell mass are relevant aspects to diabetic pathogenesis. It is important to study the regeneration of pancreatic beta cells not only to understand the mechanisms and factors involved in this process but also to provide new preventive concepts as a basis for the treatment of diabetes. Thus, these therapeutic efforts might minimize or prevent diabetes-induced oxidative stress, DNA damage, and teratogenesis.

## References

[1] American Diabetes Association (ADA) (2014). Standards of medical care in diabetes—2014. *Diabetes Care*.

[2] Bakken S, Jones DA (2006). Contributions to translational research for quality health outcomes. *Nursing Research*.

[3] National Institutes of Health Definitions under Subsection 1 (Research Objectives), Section I, (Funding Opportunity Description), Part II, (Full Text of Announcement), of RFA-RM-07-007. http://grants.nih.gov/grants/guide/rfa-files/RFA-RM-07-007.html.

[4] Rubio DM, Schoenbaum EE, Lee LS (2010). Defining translational research: implications for training. *Academic Medicine*.

[5] López-Soldado I, Herrera E (2003). Different diabetogenic response to moderate doses of streptozotocin in pregnant rats, and its long-term consequences in the offspring. *Experimental Diabesity Research*.

[6] Rudge MV, Piculo F, Marini G, Damasceno DC, Calderon IM, Barbosa AP (2013). Translational research in gestational diabetes mellitus and mild gestational hyperglycemia: current knowledge and our experience. *Arquivos Brasileiros de Endocrinologia & Metabologia*.

[7] Slack JMW (1995). Developmental biology of the pancreas. *Development*.

[8] Pan FC, Wright C (2011). Pancreas organogenesis: from bud to plexus to gland. *Developmental Dynamics*.

[9] Lacy PE (1961). Electron microscopy of the beta cell of the pancreas. *The American Journal of Medicine*.

[10] Kieffer TJ, Habener JF (1999). The glucagon-like peptides. *Endocrine Reviews*.

[11] Luft R, Efendic S, Hokfelt T, Johansson O, Arimura A (1974). Immunohistochemical evidence for the localization of somatostatin—like immunoreactivity in a cell population of the pancreatic islets. *Medical Biology*.

[12] Ekblad E, Sundler F (2002). Distribution of pancreatic polypeptide and peptide YY. *Peptides*.

[13] Prado CL, Pugh-Bernard AE, Elghazi L, Sosa-Pineda B, Sussel L (2004). Ghrelin cells replace insulin-producing *β* cells in two mouse models of pancreas development. *Proceedings of the National Academy of Sciences of the United States of America*.

[14] Assmann A, Hinault C, Kulkarni RN (2009). Growth factor control of pancreatic islet regeneration and function. *Pediatric Diabetes*.

[15] Brissova M, Fowler MJ, Nicholson WE (2005). Assessment of human pancreatic islet architecture and composition by laser scanning confocal microscopy. *Journal of Histochemistry and Cytochemistry*.

[16] Cabrera O, Berman DM, Kenyon NS, Ricordi C, Berggren P, Caicedo A (2006). The unique cytoarchitecture of human pancreatic islets has implications for islet cell function. *Proceedings of the National Academy of Sciences of the United States of America*.

[17] Orci L, Unger RH (1975). Functional subdivision of islets of Langerhans and possible role of D cells. *The Lancet*.

[18] Samols E, Bonner-Weir S, Weir GC (1986). Intra-islet insulin-glucagon-somatostatin relationships. *Clinics in Endocrinology and Metabolism*.

[19] Wieczorek G, Pospischil A, Perentes E (1998). A comparative immunohistochemical study of pancreatic islets in laboratory animals (rats, dogs, minipigs, nonhuman primates). *Experimental and Toxicologic Pathology*.

[20] Yukawa M, Takeuchi T, Watanabe T, Kitamura S (1999). Proportions of various endocrine cells in the pancreatic islets of wood mice (*Apodemus speciosus*). *Anatomia, Histologia, Embryologia*.

[21] Sujatha SR, Pulimood A, Gunasekaran S (2004). Comparative immunocytochemistry of isolated rat & monkey pancreatic islet cell types. *Indian Journal of Medical Research*.

[22] Sánchez A, Cenani S, von Lawzewitsch I (1991). Pancreatic islets in Platyrrhini monkeys: *Callithrix jacchus*, *Saimiri boliviensis*, *Aotus azarae*, and *Cebus apella*. A cytological and immunocytochemical study. *Primates*.

[23] Pipeleers D, In’t Veld P, Maes E, van de Winkel M (1982). Glucose-induced insulin release depends on functional cooperation between islet cells. *Proceedings of the National Academy of Sciences of the United States of America*.

[24] Hopcroft DW, Mason DR, Scott RS (1985). Structure-function relationships in pancreatic islets: support for intraislet modulation of insulin secretion. *Endocrinology*.

[25] Halban PA, Powers SL, George KL, Bonner-Weir S (1987). Spontaneous reassociation of dispersed adult rat pancreatic islet cells into aggregates with three-dimensional architecture typical of native islets. *Diabetes*.

[26] Bosco D, Orci L, Meda P (1989). Homologous but not heterologous contact increases the insulin secretion of individual pancreatic B-cells. *Experimental Cell Research*.

[27] Baca I, Feurle GE, Klempa I, Ziegler A, Schusdziarra V (1990). Morphometry and function of islet cells after different forms of drainage at pancreatic transplantation in rats. *European Surgical Research*.

[28] Trimble ER, Halban PA, Wollheim CB, Renold AE (1982). Functional differences between rat islets of ventral and dorsal pancreatic origin. *Journal of Clinical Investigation*.

[29] Jorns A (1994). Immunocytochemical and ultrastructural heterogeneities of normal and glibenclamide stimulated pancreatic beta cells in the rat. *Virchows Archiv*.

[30] Meda P, Denef JF, Perrelet A, Orci L (1980). Nonrandom distribution of gap junctions between pancreatic beta-cells. *The American Journal of Physiology*.

[31] Wojtusciszyn A, Armanet M, Morel P, Berney T, Bosco D (2008). Insulin secretion from human beta cells is heterogeneous and dependent on cell-to-cell contacts. *Diabetologia*.

[32] Jain R, Lammert E (2009). Cell-cell interactions in the endocrine pancreas. *Diabetes, Obesity and Metabolism*.

[33] Edvell A, Lindström P (1995). Development of insulin secretory function in young obese hyperglycemic mice (Umeå ob/ob). *Metabolism: Clinical and Experimental*.

[34] Fowden AL, Hill DJ (2001). Intra-uterine programming of the endocrine pancreas. *The British Medical Bulletin*.

[35] Pettitt DJ, Bennett PH, Saad MF, Charles MA, Nelson RG, Knowler WC (1991). Abnormal glucose tolerance during pregnancy in Pima Indian women: long-term effects on offspring. *Diabetes*.

[36] Krishnaveni GV, Veena SR, Hill JC, Kehoe S, Karat SC, Fall CHD (2010). Intrauterine exposure to maternal diabetes is associated with higher adiposity and insulin resistance and clustering of cardiovascular risk markers in Indian children. *Diabetes Care*.

[37] Portha B, Chavey A, Movassat J (2011). Early-life origins of type 2 diabetes: fetal programming of the beta-cell mass. *Experimental Diabetes Research*.

[38] Grill V, Johansson B, Jalkanen P, Eriksson UJ (1991). Influence of severe diabetes mellitus early in pregnancy in the rat: effects on insulin sensitivity and insulin secretion in the offspring. *Diabetologia*.

[39] Boloker J, Gertz SJ, Simmons RA (2002). Gestational diabetes leads to the development of diabetes in adulthood in the rat. *Diabetes*.

[40] Van Assche FA, Aerts L, Holemans K (1991). The effects of maternal diabetes on the offspring. *Bailliere’s Clinical Obstetrics and Gynaecology*.

[41] Lucas A (1998). Programming by early nutrition: an experimental approach. *Journal of Nutrition*.

[42] Aguayo-Mazzucato C, Sanchez-Soto C, Godinez-Puig V, Gutiérrez-Ospina G, Hiriart M (2006). Restructuring of pancreatic islets and insulin secretion in a postnatal critical window. *PLoS ONE*.

[43] Fowden AL, Ward JW, Wooding FPB, Forhead AJ, Constancia M (2006). Programming placental nutrient transport capacity. *Journal of Physiology*.

[44] Lamberts SWJ, Koper JW, Reubi JC (1987). Potential role of somatostatin analogues in the treatment of cancer. *European Journal of Clinical Investigation*.

[45] Setyono-Han B, Henkelman MS, Foekens JA, Klijn JGM (1987). Direct inhibitory effects of somatostatin (analogues) on the growth of human breast cancer cells. *Cancer Research*.

[46] Nobuhiko T, Miho N, Mitsuko F (2011). Octreotide-treated diabetes accompanied by endogenous hyperinsulinemic hypoglycemia and protein-losing gastroenteropathy. *Case Reports in Medicine*.

[47] Aerts L, van Assche FA (1992). Islet transplantation in diabetic pregnant rats normalizes glucose homeostasis in their offspring. *Journal of Developmental Physiology*.

[48] de Gasparo M, Milner RDG (1980). The timing of fetal B cell hyperplasia in diabetic rat pregnancy. *Diabetologia*.

[49] Ryan EA, Liu D, Bell RC, Finegood DT, Crawford J (1995). Long term consequences in offspring of diabetes in pregnancy: studies with syngeneic islet-transplanted streptozotocin-diabetic rats. *Endocrinology*.

[50] Han J, Xu J, Long YS, Epstein PN, Liu YQ (2007). Rat maternal diabetes impairs pancreatic *β*-cell function in the offspring. *The American Journal of Physiology—Endocrinology and Metabolism*.

[51] Zeng CJ, Zhang L, Yang HX (2010). Effects of severe hyperglycaemia in pregnancy and early overfeeding on islet development and insulin resistance. *Zhonghua Fu Chan Ke Za Zhi*.

[52] Marchand KC, Arany EJ, Hill DJ (2010). Effects of atorvastatin on the regeneration of pancreatic *β*-cells after streptozotocin treatment in the neonatal rodent. *The American Journal of Physiology—Endocrinology and Metabolism*.

[53] Branco RCS, de Oliveira JC, Grassiolli S (2012). Maternal protein malnutrition does not impair insulin secretion from pancreatic islets of offspring after transplantation into diabetic rats. *PLoS ONE*.

[54] Aref AB, Ahmed OM, Ali LA, Semmler M (2013). Maternal rat diabetes mellitus deleteriously affects insulin sensitivity and beta-cell function in the offspring. *Journal of Diabetes Research*.

[55] Iessi IL, Sinzato YK, Calderon IMP, Rudge MVC, Damasceno DC Pancreatic islet hormonal triad in diabetic pregnant rats and their offspring.

[56] Evans JL, Maddux BA, Goldfine ID (2005). The molecular basis for oxidative stress-induced insulin resistance. *Antioxidants and Redox Signaling*.

[57] Folli F, Corradi D, Fanti P (2011). The role of oxidative stress in the pathogenesis of type 2 diabetes mellitus micro-and macrovascular complications: avenues for a mechanistic-based therapeutic approach. *Current Diabetes Reviews*.

[58] Kim EK, Choi EJ (2010). Pathological roles of MAPK signaling pathways in human diseases. *Biochimica et Biophysica Acta—Molecular Basis of Disease*.

[59] Sigala I, Zacharatos P, Toumpanakis D (2011). MAPKs and NF-*κ*B differentially regulate cytokine expression in the diaphragm in response to resistive breathing: the role of oxidative stress. *The American Journal of Physiology—Regulatory Integrative and Comparative Physiology*.

[60] Muscogiuri G, Salmon AB, Aguayo-Mazzucato C (2013). Genetic disruption of SOD1 gene causes glucose intolerance and impairs *β*-cell function. *Diabetes*.

[61] National Diabetes Data Group (1979). Classification and diagnosis of diabetes and other categories of glucose intolerance. *Diabetes*.

[62] WHO Expert Committee on Diabetes Mellitus (1980). *Technical Report Series 646, Second Report*.

[63] Kahn R (1997). Report of the expert committee on the diagnosis and classification of diabetes mellitus. *Diabetes Care*.

[64] World Health Organisation (1999). *Definition, Diagnosis, and Classification of Diabetes Mellitus and Its Complications. Report of a WHO Consultation—Part 1: Diagnosis and Classification of Diabetes Mellitus*.

[65] Unwin N, Shaw J, Zimmet P, Alberti KGMM (2002). Impaired glucose tolerance and impaired fasting glycaemia: the current status on definition and intervention. *Diabetic Medicine*.

[66] Ward DT, Yau SK, Mee AP (2001). Functional, molecular, and biochemical characterization of streptozotocin-induced diabetes. *Journal of the American Society of Nephrology*.

[67] Lenzen S (2008). The mechanisms of alloxan- and streptozotocin-induced diabetes. *Diabetologia*.

[68] Portha B, Levacher C, Picon L, Rosselin G (1974). Diabetogenic effect of streptozotocin in the rat during the perinatal period. *Diabetes*.

[69] Lenzen S, Tiedge M, Jörns A, Munday R, Shafrir E (1996). Alloxan derivatives as a tool for the elucidation of the mechanism of the diabetogenic action of alloxan. *Lessons from Animal Diabetes*.

[70] Arison RN, Ciaccio EI, Glitzer MS, Cassaro JA, Pruss MP (1967). Light and electron microscopy of lesions in rats rendered diabetic with streptozotocin. *Diabetes*.

[71] Eriksson UJ, Borg LAH, Cederberg J (2000). Pathogenesis of diabetes-induced congenital malformations. *Upsala Journal of Medical Sciences*.

[72] Eriksson UJ, Cederberg J, Wentzel P (2003). Congenital malformations in offspring of diabetic mothers—animal and human studies. *Reviews in Endocrine and Metabolic Disorders*.

[73] Damasceno DC, Volpato GT, Ide MC, Aguilar R, Rudge MVC (2004). Effect of Bauhinia forficata extract in diabetic pregnant rats: maternal repercussions. *Phytomedicine*.

[74] Rudge MVC, Damasceno DC, Volpato GT, Almeida FCG, Calderon IMP, Lemonica IP (2007). Effect of Ginkgo biloba on the reproductive outcome and oxidative stress biomarkers of streptozotocin-induced diabetic rats. *Brazilian Journal of Medical and Biological Research*.

[75] Volpato GT, Damasceno DC, Rudge MVC, Padovani CR, Calderon IMP (2008). Effect of Bauhinia forficata aqueous extract on the maternal-fetal outcome and oxidative stress biomarkers of streptozotocin-induced diabetic rats. *Journal of Ethnopharmacology*.

[76] de Souza Mda S, Lima PHO, Sinzato YK, Rudge MVC, Pereira OCM, Damasceno DC (2009). Effects of cigarette smoke exposure on pregnancy outcome and offspring of diabetic rats. *Reproductive BioMedicine Online*.

[77] de Souza Mda S, Sinzato YK, Lima PHO, Calderon IMP, Rudge MVC, Damasceno DC (2010). Oxidative stress status and lipid profiles of diabetic pregnant rats exposed to cigarette smoke. *Reproductive BioMedicine Online*.

[78] Tsuji K, Taminato T, Usami M (1988). Characteristic features of insulin secretion in the streptozotocin-induced NIDDM rat model. *Metabolism: Clinical and Experimental*.

[79] Merzouk H, Madani S, Sari DC, Prost J, Bouchenak M, Belleville J (2000). Time course of changes in serum glucose, insulin, lipids and tissue lipase activities in macrosomic offspring of rats with streptozotocin-induced diabetes. *Clinical Science*.

[80] Sinzato YK, Lima PH, de Campos KE, Kiss ACI, Rudge MVC, Damascene DC (2009). Neonatally-induced diabetes: lipid profile outcomes and oxidative stress status in adult rats. *Revista da Associacao Medica Brasileira*.

[81] Damasceno DC, Kiss ACI, Sinzato YK (2011). Maternal-fetal outcome, lipid profile and oxidative stress of diabetic rats neonatally exposed to streptozotocin. *Experimental and Clinical Endocrinology and Diabetes*.

[82] Uriu-Hare JY, Keen CL, Applegate EA, Stern JS (1989). The influence of moderate exercise in diabetic and normal pregnancy on maternal and fetal outcome in the rat. *Life Sciences*.

[83] Bueno A, Sinzato YK, Sudano MJ (2014). Short and long-term repercussions of the experimental diabetes in embryofetal development. *Diabetes/Metabolism Research and Reviews*.

[84] Damasceno DC, Silva HP, Vaz GF (2013). Diabetic rats exercised prior to and during pregnancy: maternal reproductive outcome, biochemical profile, and frequency of fetal anomalies. *Reproductive Sciences*.

[85] Saito FH, Damasceno DC, Dallaqua B (2013). Heat shock protein production and immunity and altered fetal development in diabetic pregnant rats. *Cell Stress and Chaperones*.

[86] Lima PHO, Sinzato YK, Gelaleti RB, Calderon IMP, Rudge MVC, Damasceno DC (2012). Genotoxicity evaluation in severe or mild diabetic pregnancy in laboratory animals. *Experimental and Clinical Endocrinology and Diabetes*.

[87] Piculo F, Marini G, Barbosa AM (2014). Urethral striated muscle and extracellular matrix morphological characteristics among mildly diabetic pregnant rats: translational approach. *International Urogynecology Journal*.

[88] Kiss ACI, Woodside B, Sinzato YK (2013). Neonatally induced mild diabetes: influence on development, behavior and reproductive function of female Wistar rats. *Diabetology and Metabolic Syndrome*.

[89] Spada AP, Damasceno DC, Sinzato YK (2014). Oxidative stress in maternal blood and placenta from mild diabetic rats. *Reproductive Sciences*.

[90] Dallaqua B, Saito FH, Rodrigues T (2013). *Azadirachta indica* treatment on the congenital malformations of fetuses from rats. *Journal of Ethnopharmacology*.

[91] Dallaqua B, Saito FH, Rodrigues T (2012). Treatment with *Azadirachta indica* in diabetic pregnant rats: negative effects on maternal outcome. *Journal of Ethnopharmacology*.

[92] Kiss ACI, Woodside B, Felício LF, Anselmo-Franci J, Damasceno DC (2012). Impact of maternal mild hyperglycemia on maternal care and offspring development and behavior of Wistar rats. *Physiology and Behavior*.

[93] Sinzato YK, Volpato GT, Iessi IL (2012). Neonatally induced mild diabetes in rats and its effect on maternal, placental, and fetal parameters. *Experimental Diabetes Research*.

[94] Georgia S, Bhushan A (2004). *β* cell replication is the primary mechanism for maintaining postnatal *β* cell mass. *Journal of Clinical Investigation*.

[95] Liang XD, Guo YY, Sun M (2011). Streptozotocin-induced expression of Ngn3 and Pax4 in neonatal rat pancreatic *α*-cells. *World Journal of Gastroenterology*.

[96] Goss RJ, Falkner F, Tanner JM (1986). Modes of growth and regeneration: mechanisms, regulation, distribution. *Human Growth: A Comprehensive Treatise*.

[97] Bonner-Weir S, Trent DF, Honey RN, Weir GC (1981). Responses of neonatal rat islets to streptozotocin: limited B-cell regeneration and hyperglycemia. *Diabetes*.

[98] Bonner-Weir S, Weir GC (2005). New sources of pancreatic *β*-cells. *Nature Biotechnology*.

[99] Minami K, Seino S (2008). Regeneration of the pancreas. *Nippon Rinsho*.

[100] Collombat P, Mansouri A, Hecksher-Sørensen J (2003). Opposing actions of Arx and Pax4 in endocrine pancreas development. *Genes and Development*.

[101] Scaglia L, Cahill CJ, Finegood DT, Bonner-Weir S (1997). Apoptosis participates in the remodeling of the endocrine pancreas in the neonatal rat. *Endocrinology*.

[102] Oppenheim RW (1991). Cell death during development of the nervous system. *Annual Review of Neuroscience*.

[103] Coles HSR, Burne JF, Raff MC (1993). Large-scale normal cell death in the developing rat kidney and its reduction by epidermal growth factor. *Development*.

[104] Terada T, Nakanuma Y (1995). Detection of apoptosis and expression of apoptosis-related proteins during human intrahepatic bile duct development. *The American Journal of Pathology*.

[105] Blaschke AJ, Staley K, Chun J (1996). Widespread programmed cell death in proliferative and postmitotic regions of the fetal cerebral cortex. *Development*.

[106] Portha B, Blondel O, Serradas P (1989). The rat models of non-insulin dependent diabetes induced by neonatal streptozotocin. *Diabete et Metabolisme*.

[107] Bonner-Weir S, Deery D, Leahy JL, Weir GC (1989). Compensatory growth of pancreatic *β*-cells in adult rats after short-term glucose infusion. *Diabetes*.

[108] Bonner-Weir S (1994). Regulation of pancreatic *β*-cell mass in vivo. *Recent Progress in Hormone Research*.

[109] Gradwohl G, Dierich A, LeMeur M, Guillemot F (2000). Neurogenin3 is required for the development of the four endocrine cell lineages of the pancreas. *Proceedings of the National Academy of Sciences of the United States of America*.

[110] Jenny M, Uhl C, Roche C (2002). Neurogenin3 is differentially required for endocrine cell fate specification in the intestinal and gastric epithelium. *The EMBO Journal*.

[111] Gu G, Brown JR, Melton DA (2003). Direct lineage tracing reveals the ontogeny of pancreatic cell fates during mouse embryogenesis. *Mechanisms of Development*.

[112] Li SW, Koya V, Li Y (2010). Pancreatic duodenal homeobox 1 protein is a novel *Β*-cell-specific autoantigen for type I diabetes. *Laboratory Investigation*.

[113] Smith SB, Ee HC, Conners JR, German MS (1999). Paired-homeodomain transcription factor PAX4 acts as a transcriptional repressor in early pancreatic development. *Molecular and Cellular Biology*.

[114] Dohrmann C, Gruss P, Lemaire L (2000). Pax genes and the differentiation of hormone-producing endocrine cells in the pancreas. *Mechanisms of Development*.

[115] Courtney M, Gjernes E, Druelle N (2013). The inactivation of Arx in pancreatic *α*-cells triggers their neogenesis and conversion into functional *β*-like cells. *PLoS Genetics*.

[116] García DG, García DR (2009). Avances en la patogénesis de la embriopatía diabética. *Revista Médica de Chile*.

[117] Moore TR, Creasy RK, Resnik R (1999). Diabetes in pregnancy. *Maternal Fetal Medicine: Principles and Practice*.

[118] Eriksson UJ (2009). Congenital anomalies in diabetic pregnancy. *Seminars in Fetal and Neonatal Medicine*.

[119] Zabihi S, Loeken MR (2010). Understanding diabetic teratogenesis: where are we now and where are we going?. *Birth Defects Research A: Clinical and Molecular Teratology*.

[120] White P (1949). Pregnancy complicating diabetes. *The American Journal of Medicine*.

[121] Freinkel N, Ogata E, Metzger BE, Rifkin H, Porte D (1990). The offspring of the mother with diabetes. *Diabetes Mellitus: Theory and Practice*.

[122] Fuhrmann K, Reiher H, Semmler K, Glöckner E (1984). The effect of intensified conventional insulin therapy before and during pregnancy on the malformation rate in offspring of diabetic mothers. *Experimental and Clinical Endocrinology*.

[123] Cohen MM, Shiota K (2002). Teratogenesis of holoprosencephaly. *The American Journal of Medical Genetics*.

[124] Correa A, Gilboa SM, Besser LM (2008). Diabetes mellitus and birth defects. *The American Journal of Obstetrics and Gynecology*.

[125] Calciu C, Kubow S, Chan HM (2002). Interactive dysmorphogenic effects of toxaphene or toxaphene congeners and hyperglycemia on cultured whole rat embryos during organogenesis. *Toxicology*.

[126] Damasceno DC, Volpato GT, Calderon IMP, Rudge MVC (2002). Oxidative stress and diabetes in pregnant rats. *Animal Reproduction Science*.

[127] Gäreskog M, Cederberg J, Eriksson UJ, Wentzel P (2007). Maternal diabetes in vivo and high glucose concentration in vitro increases apoptosis in rat embryos. *Reproductive Toxicology*.

[128] Hod M, Star S, Passonneau J, Unterman TG, Freinkel N (1990). Glucose-induced dysmorphogenesis in the cultured rat conceptus: prevention by supplementation with myo-inositol. *Israel Journal of Medical Sciences*.

[129] Eriksson UJ, Naeser P, Brolin SE (1986). Increased accumulation of sorbitol in offspring of manifest diabetic rats. *Diabetes*.

[130] Pinter E, Reece EA, Leranth CZ (1986). Yolk sac failure in embryopathy due to hyperglycemia: ultrastructural analysis of yolk sac differentiation associated with embryopathy in rat conceptuses under hyperglycemic conditions. *Teratology*.

[131] Goldman AS, Goto MP (1991). Biochemical basis of the diabetic embryopathy. *Israel Journal of Medical Sciences*.

[132] Hagay ZJ, Weiss Y, Zusman I (1995). Prevention of diabetes-associated embryopathy by overexpression of the free radical scavenger copper zinc superoxide dismutase in transgenic mouse embryos. *The American Journal of Obstetrics and Gynecology*.

[133] Herrera E (2002). Implications of dietary fatty acids during pregnancy on placental, fetal and postnatal development—a review. *Placenta*.

[134] Reece EA, Wu YK, Zhao Z, Dhanasekaran D (2006). Dietary vitamin and lipid therapy rescues aberrant signaling and apoptosis and prevents hyperglycemia-induced diabetic embryopathy in rats. *The American Journal of Obstetrics and Gynecology*.

[135] Cha YI, Solnica-Krezel L, DuBois RN (2006). Fishing for prostanoids: deciphering the developmental functions of cyclooxygenase-derived prostaglandins. *Developmental Biology*.

[136] Piddington R, Joyce J, Dhanasekaran P, Baker L (1996). Diabetes mellitus affects prostaglandin E2 levels in mouse embryos during neurulation. *Diabetologia*.

[137] Wentzel P, Welsh N, Eriksson UJ (1999). Developmental damage, increased lipid peroxidation, diminished cyclooxygenase-2 gene expression, and lowered prostaglandin E2 levels in rat embryos exposed to a diabetic environment. *Diabetes*.

[138] Akazawa S (2005). Diabetic embryopathy: studies using a rat embryo culture system and an animal model. *Congenital Anomalies*.

[139] Brownlee M (2001). Biochemistry and molecular cell biology of diabetic complications. *Nature*.

[140] El-Hage S, Singh SM (1990). Temporal expression of genes encoding free radical-metabolizing enzymes is associated with higher mRNA levels during in utero development in mice. *Developmental Genetics*.

[141] Eriksson UJ, Borg LAH (1993). Diabetes and embryonic malformations: role of substrate-induced free-oxygen radical production for dysmorphogenesis in cultured rat embryos. *Diabetes*.

[142] Kinalski M, Sledziewski A, Telejko B, Kowalska I, Kretowski A, Kinalska I (2001). Evaluation of lipid peroxidation and acid-base status in cord blood of newborns after diabetes in pregnancy. *Przeglad Lekarski*.

[143] Ornoy A (2012). Biomarkers of maternal diabetes and its complication in pregnancy. *Reproductive Toxicology*.

[144] Wentzel P, Eriksson UJ (2011). Altered gene expression in rat cranial neural crest cells exposed to a teratogenic glucose concentration in vitro-paradoxical downregulation of antioxidative defense genes. *Birth Defects Research B—Developmental and Reproductive Toxicology*.

[145] Emerit I (1994). Reactive oxygen species, chromosome mutation, and cancer: possible role of clastogenic factors in carcinogenesis. *Free Radical Biology and Medicine*.

[146] Emerit I (1990). Clastogenic factors: detection and assay. *Methods in Enzymology*.

[147] Young ID, Brock DJH, Rodeck CH, Ferguson-Smith MA (1992). Congenital malformations: incidence and genetics of congenital malformations. *Prenatal Diagnosis and Screening*.

[148] Breen AP, Murphy JA (1995). Reactions of oxyl radicals with DNA. *Free Radical Biology and Medicine*.

[149] Marnett LJ (2000). Oxyradicals and DNA damage. *Carcinogenesis*.

[150] Newcombe TG, Loeb LA, Aruoma OI, Halliwell B (1998). Mechanism of mutagenicity of oxidatively-modified bases. *Molecular Biology of Free Radicals in Human Disease*.

[151] Azqueta A, Shaposhnikov S, Collins AR (2009). DNA oxidation: investigating its key role in environmental mutagenesis with the comet assay. *Mutation Research—Genetic Toxicology and Environmental Mutagenesis*.

[152] Loft S, Vistisen K, Ewertz M, Tjonneland A, Overvad K, Poulsen HE (1992). Oxidative DNA damage estimated by 8-hydroxydeoxyguanosine excretion in humans: influence of smoking, gender and body mass index. *Carcinogenesis*.

[153] Erhola M, Toyokuni S, Okada K (1997). Biomarker evidence of DNA oxidation in lung cancer patients: association of urinary 8-hydroxy-2′-deoxyguanosine excretion with radiotherapy, chemotherapy, and response to treatment. *FEBS Letters*.

[154] Qiu C, Hevner K, Abetew D, Enquobahrie DA, Williams MA (2011). Oxidative DNA damage in early pregnancy and risk of gestational diabetes mellitus: a pilot study. *Clinical Biochemistry*.

[155] Collins AR, Duthie SJ, Dobson VL (1993). Direct enzymic detection of endogenous oxidative base damage in human lymphocyte DNA. *Carcinogenesis*.

[156] Collins AR, Dušinská M, Gedik CM, Štětina R (1996). Oxidative damage to DNA: do we have a reliable biomarker?. *Environmental Health Perspectives*.

[157] Mossman BT, Ireland CM, Filipak M, LeDoux S, Wilso GL (1986). Comparative interactions of streptozotocin and chlorozotocin with DNA of an insulin-secreting cell line (RINr). *Diabetologia*.

[158] Pettepher CC, LeDoux SP, Bohr VA, Wilson GL (1991). Repair of alkali-labile sites within the mitochondrial DNA of RINr 38 cells after exposure to the nitrosourea streptozotocin. *The Journal of Biological Chemistry*.

[159] Damasceno DC, Volpato GT, Sinzato YK (2011). Genotoxicity and fetal abnormality in streptozotocin-induced diabetic rats exposed to cigarette smoke prior to and during pregnancy. *Experimental and Clinical Endocrinology and Diabetes*.

